# Diagnostic value of late gadolinium enhancement at cardiovascular magnetic resonance to distinguish arrhythmogenic right ventricular cardiomyopathy from differentials

**DOI:** 10.1016/j.jocmr.2024.101059

**Published:** 2024-07-08

**Authors:** Lian Y. Rekker, Steven A. Muller, Alessio Gasperetti, Mimount Bourfiss, Marish I.F.J. Oerlemans, Maarten J. Cramer, Stefan L. Zimmerman, Dennis Dooijes, Hanke Schalkx, Pim van der Harst, Cynthia A. James, J. Peter van Tintelen, Marco Guglielmo, Birgitta K. Velthuis, Anneline S.J.M. te Riele

**Affiliations:** a Department of Cardiology, University Medical Center Utrecht, Utrecht, the Netherlands; b Netherlands Heart Institute, Utrecht, the Netherlands; c Division of Medicine, Department of Cardiology, Johns Hopkins University, Baltimore, Maryland, USA; d Department of Clinical Electrophysiology & Cardiac Pacing, Centro Cardiologico Monzino, IRCCS, Milano, Italy; e Department of Radiology, Johns Hopkins University, Baltimore, Maryland, USA; f Department of Genetics, University Medical Center Utrecht, Utrecht University, Utrecht, the Netherlands; g Department of Radiology, University Medical Center Utrecht, Utrecht, the Netherlands

**Keywords:** ARVC, LGE, Padua criteria, Magnetic resonance imaging, Delayed enhancement

## Abstract

**Background:**

While late gadolinium enhancement (LGE) is proposed as a diagnostic criterion for arrhythmogenic right ventricular cardiomyopathy (ARVC), the potential of LGE to distinguish ARVC from differentials remains unknown. We aimed to assess the diagnostic value of LGE for ARVC diagnosis.

**Methods:**

We included 132 subjects (60% male, 47 ± 11 years) who had undergone cardiac magnetic resonance imaging with LGE assessment for ARVC or ARVC differentials. ARVC was diagnosed as per 2010 Task Force Criteria (n = 55). ARVC differentials consisted of familial/genetic dilated cardiomyopathy (n = 25), myocarditis (n = 13), sarcoidosis (n = 20), and amyloidosis (n = 19). The diagnosis of all differentials was based on the most current standard of reference. The presence of LGE was evaluated using a 7-segment right ventricle (RV) and 17-segment left ventricle (LV) model. Subsequently, we assessed LGE patterns for every patient individually for fulfilling LV- and/or RV-LGE per Padua criteria, independent of their clinical diagnosis (i.e. phenotype). Diagnostic values were analyzed using sensitivity and specificity for any RV-LGE, any LV-LGE, RV-LGE per Padua criteria, and prevalence graphs for LV-LGE per Padua criteria. The optimal integration of LGE for ARVC diagnosis was determined using classification and regression tree analysis.

**Results:**

One-third (38%) of ARVC patients had RV-LGE, while half (51%) had LV-LGE. RV-LGE was less frequently observed in ARVC vs non-ARVC patients (38% vs 58%, p = 0.034) leading to a poor discriminatory potential (any RV-LGE: sensitivity 38%, specificity 42%; RV-LGE per Padua criteria: sensitivity 36%, specificity 44%). Compared to ARVC patients, non-ARVC patients more often had LV-LGE (91% vs 51%, p < 0.001) which was also more globally distributed (median 9 [interquartile range (IQR): 3–13] vs 0 [IQR: 0–3] segments, p < 0.001). The absence of anteroseptal and absence of extensive (≥5 segments) mid-myocardial LV-LGE, and absence of moderate (≥2 segments) mid-myocardial LV-LGE predicted ARVC with good diagnostic performance (sensitivity 93%, specificity 78%).

**Conclusion:**

LGE is often present in ARVC differentials and may lead to false positive diagnoses when used without knowledge of LGE patterns. Moderate RV-LGE without anteroseptal and mid-myocardial LV-LGE is typically observed in ARVC.

## Graphical abstract

**Figure:**
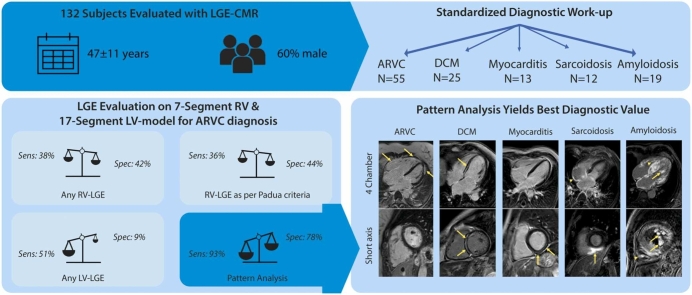

## 1 Background

Arrhythmogenic right ventricular cardiomyopathy (ARVC) is a highly arrhythmogenic disease characterized by cardiomyocyte loss and fibrofatty replacement of predominantly the right ventricle (RV) [1]. This traditional disease phenotype was based on autopsy studies and predated the widespread use of genetic testing and non-invasive tissue characterization [2]. The genetic era led to identification of asymptomatic relatives with less severe disease expression and an appreciation of distinct genotype-phenotype correlations [3], [4]. In addition, cardiovascular magnetic resonance (CMR) imaging using late gadolinium enhancement (LGE) has shown that ARVC not only affects the RV but can also lead to (sometimes predominant) left ventricular (LV) involvement [5]. Both these genetic and CMR insights resulted in the new designation of “Arrhythmogenic Cardiomyopathy” (ACM), which represents a modern concept of genetically or systemically determined biventricular heart diseases (e.g. ARVC, sarcoidosis, myocarditis, and amyloidosis) that predisposes the patient to global and/or regional dysfunction and arrhythmias [6].

The clinical gold standard for ARVC diagnosis is the consensus-based “Task Force Criteria” (TFC) [7]. Although tissue characteristics are included in the TFC, current criteria can be met only with endomyocardial biopsy, which is invasive and rarely used clinically, and do not include non-invasive imaging criteria, such as fatty or fibrotic replacement of the myocardium. A potential new framework for diagnosis of the ACM disease spectrum includes criteria for right-sided and left-sided disease [8]. These “Padua criteria” make use of tissue characterization by LGE-CMR to determine fibrofatty replacement in both the LV and RV. While this makes sense from a histopathological standpoint, LGE is a qualitative parameter that can be misinterpreted if not evaluated by an experienced reviewer, particularly in the thin RV wall. In addition, LGE can be observed in many differential diagnoses, such as dilated cardiomyopathy (DCM), (resolved) myocarditis, sarcoidosis, and amyloidosis [9], [10]. A significant overlap with these diseases and a large number of false positive diagnoses may therefore be at risk. Furthermore, the diagnostic value of LGE for ARVC within the spectrum of ARVC differentials has never been systematically evaluated. Therefore, the aim of this study was to determine the potential of LGE to distinguish ARVC from its differentials.

## 2 Methods

### 2.1 Study population and study design

This was a retrospective study embedded in the UNRAVEL biobank [11] (www.unravelrdp.nl). From this biobank, we included patients who were evaluated for cardiomyopathy at our hospital including a CMR of sufficient quality for analysis with long-axis and short-axis planes covering the entire LV and RV. Patient records were filtered for the clinical diagnoses of interest (i.e. ARVC, DCM, (resolved) myocarditis, sarcoidosis, and amyloidosis), which were subsequently checked by independent experienced observers as described below. The study protocol was exempt from the Medical Research Involving Human Subjects Act (WMO), as determined by the medical ethical committee at our hospital (protocol number 18-336/C).

### 2.2 Diagnostic classification

Diagnostic classification (i.e. phenotyping) was based on predefined strict criteria, as described below. Diagnoses were first adjudicated based on medical record files by a single independent observer (L.R.) and subsequently confirmed by two experienced observers (S.M. and A.T.R.) who independently reviewed all raw test results and clinic notes to verify the diagnosis.

ARVC patients (n = 55) were included if they fulfilled definite diagnosis as per 2010 TFC (i.e. in the presence of two major, one major plus two minor, or four minor diagnostic criteria) [7]. In addition, to avoid potential overlap with ARVC differentials, we limited inclusion to those who harbored a (likely) pathogenic variant in a gene that was considered strongly or moderately associated with ARVC (n = 40) [12] or to those who did not require family history to fulfill definite ARVC diagnosis (n = 15), as specified in Additional file 1: Table S1.

DCM patients (n = 25) were diagnosed based on the World Health Organization criteria as LV or biventricular systolic dysfunction (i.e. LV ejection fraction [EF] <45%) and dilatation (i.e. >2 standard deviations from age- and sex-specific normal values corrected for body surface area) that is not explained by abnormal loading conditions or coronary artery disease [13]. We required either the presence of a (likely) pathogenic variant in a gene that was considered strongly or moderately associated with DCM (n = 16) [14], and/or presence of familial DCM as per Mestroni criteria (n = 9) [15] to avoid overlap with other heart diseases in an end stage (detailed information provided in Additional file 1: Table S1).

(Resolved) myocarditis (n = 13) was diagnosed based on the history of a distinct viral episode with the fulfillment of histopathologic Dallas criteria in myocardial tissue (n = 4) [16], or (in the absence of myocardial biopsy) based on the European Society of Cardiology (ESC) position statement with a strongly suspected or confirmed offending microbial cause (n = 9) [17]. Of note, while genetic testing was not routinely performed in this patient subgroup, none of the (resolved) myocarditis patients had a family history of cardiomyopathy or unexplained sudden cardiac arrest.

Sarcoidosis patients (n = 20) were diagnosed as per Heart Rhythm Society (HRS) Expert Consensus Statement [18]: diagnosis was based on the presence of non-caseating granulomas on histopathological examination of myocardial tissue (n = 3); or, in the absence of an endomyocardial biopsy, on the combination of histologically confirmed extra-cardiac sarcoidosis plus evidence of cardiac involvement (n = 17).

Cardiac amyloidosis patients (n = 19) included both amyloid light chain (AL; n = 10) and amyloid transthyretin (ATTR; n = 9) amyloidosis and were diagnosed based on the 2021 ESC position paper [19]. In short, as per ESC framework and following the Utrecht Amyloidosis clinical pathway [20], ATTR amyloidosis patients were diagnosed by either bone scintigraphy in the absence of monoclonal antibodies (n = 7) or by endomyocardial biopsy positive for transthyretin (n = 2); patients with AL amyloidosis were diagnosed based on either endomyocardial biopsy positive for kappa or lambda (n = 1) or extra-cardiac biopsy positive for kappa or lambda with evidence of cardiac involvement (n = 9).

Of note, for all specific subgroups, we required that any other diagnoses were reasonably excluded based on tests ordered by the discretion of the treating physician. In addition, CMR-based criteria were disregarded from all the above-mentioned definitions, to avoid a circular argument in which CMR-based criteria would predict themselves. Of note, all patients were diagnosed before or at the time of CMR.

### 2.3 Clinical data collection

Data were retrospectively collected from medical records. All patients underwent a full cardiomyopathy workup, including 12-lead electrocardiogram (ECG), echocardiography, and CMR imaging. Other tests including (but not limited to) genetic testing, Holter monitoring, cardiac computed tomography, positron-emission tomography, and coronary angiography were performed upon discretion of the treating physician. Given the focus of this manuscript on CMR results, we will not further explore these additional testing results apart from their role in establishing the diagnosis of interest.

### 2.4 CMR acquisition and analysis

All CMRs were acquired on a 1.5T or 3T scanner (Philips, Medical Systems, Best, the Netherlands) with a phased array cardiac coil during repeated end-expiratory breath holds. ECG-gated cine images, fast spin-echo images, and contrast-enhanced images after administration of a gadolinium chelate were acquired in both long-axis and short-axis planes covering the entire RV and LV. Locally available software was used for semi-automatic analysis of biventricular EF, end-diastolic volume, and end-systolic volume on short-axis views (Extended MR-WorkSpace, [Philips Medical Systems, Eindhoven, the Netherlands] or QMass [(Medis Medical Imaging Systems, Leiden, the Netherlands]). Dimensions were indexed to body surface area using the DuBois formula.

The presence and transmurality of LGE on a phase-sensitive inversion recovery sequence was visually evaluated by an experienced cardiovascular radiologist (B.V.) and scored on a 7-segment RV and 17-segment LV model, for every segment separately (for segmentation used in this study see Additional file 1: Fig. S1). Consequently, any left ventricular LGE (LV-LGE) and any right ventricular LGE (RV-LGE) corresponded to LGE present in segments 1–17 and segments 18–24, respectively. Degrees of LGE transmurality were evaluated for the LV only, given the well-recognized difficulty of assessing degrees of transmurality in the thin RV wall [8]. In addition, the observers also assessed the “Whale’s Tail” sign, which was defined as subepicardial LGE from the LV extending into the septal wall and RV and is previously reported as a specific sign for sarcoidosis [21] (Additional file 1: Fig. S2). Of note, given the frequent finding of insertion fibrosis (i.e. LGE in the septum at the RV insertion points) in healthy subjects [22], we disregarded this as an abnormal finding for the purpose of this study.

Intra- and interobserver reproducibilities were assessed as described below.

### 2.5 Padua criteria

Using the above-mentioned LGE evaluation, patients were subsequently assessed for LGE using the Padua criteria algorithm for both the LV and RV, independent of their clinical diagnosis (i.e. phenotype). Within this framework [8], RV-LGE per Padua criteria is considered present if observed in the RV inlet, outlet, or apex in two orthogonal views (i.e. LGE present in at least one of the following segments 18–20, 23, or 24); while LV-LGE per Padua criteria is considered present if observed in the subepicardial or mid-myocardial free wall, septum or both excluding septal junctional LGE and observed in two orthogonal views (i.e. subepicardial/mid-myocardial LGE in at least one of the following segments 1–16) (for segmentation see Additional file 1: Fig. S1). Consequently, a patient can fulfill (1) both LV and RV-LGE per Padua criteria; (2) only LV-LGE per Padua criteria; (3) only RV-LGE per Padua criteria; or (4) neither fulfill LV nor RV-LGE per Padua criteria.

### 2.6 Statistical analysis

Nominal variables were expressed as numbers (%), and continuous variables as mean ± standard deviation or median [interquartile range], as appropriate. Comparisons for binary variables were performed by chi-square or Fisher’s exact test. For continuous variables, we used the independent Student t-test, one-way analysis of variance, Mann-Whitney U test, or Kruskal-Wallis test. The presence, distribution, and transmurality of LV-LGE and the presence and distribution of RV-LGE were tested by comparing the ARVC group with all differentials separately, as well as with the combined “non-ARVC” group.

We subsequently calculated conventional diagnostic metrics (i.e. sensitivity, specificity, and accuracy) and evaluated the reproducibility for “any RV-LGE,” “any LV-LGE,” and “RV-LGE per Padua criteria” to distinguish ARVC from its differentials. Since the 2010 TFC did not include diagnostic criteria for LV-dominant subtypes and the Padua criteria cannot serve as a gold standard for itself, conventional diagnostic metrics cannot be reliably calculated. Consequently, we reverted to reporting the prevalence of “LV-LGE per Padua criteria” per differential. Reproducibility was determined through intra- and interobserver variabilities. Intra-observer variability was assessed by re-assessing 30 randomly selected subjects by the first observer. For interobserver variability, the same 30 selected subjects were re-assessed by a second observer, independent of the first observer.

Subsequently, we repeated the above-mentioned evaluation on the diagnostic value of LGE with subjects harboring a (likely) pathogenic variant (i.e. “genetically proven ARVC”) and compared those separately to the ARVC differentials.

As a proof of concept to evaluate if there is diagnostic value in LGE, we performed classification and regression tree (CART)-analysis to determine which LGE parameters were most predictive for ARVC by comparing the ARVC group with the non-ARVC group. CART is an empirical non-parametric technique that produces a decision tree by a series of binary splits (recursive partitioning) of the data. We evaluated all RV- and LV-LGE parameters (both extent and distribution) in the CART model. The model subsequently selects variables in order of magnitude of improvement in prediction of the outcome, and the variable that contributes most to the outcome is listed at the top of the tree. A 10-fold cross-validation was used to select the best tree with the lowest error estimate.

A p-value <0.05 was considered statistically significant throughout; however, given the large number of comparisons, Bonferroni corrections were added to limit the probability of a type I statistical error. The statistically significant p-value after Bonferroni correction is noted in the footnote of each table. Reproducibility was measured by Cohen's Kappa-coefficient with cut-offs as previously suggested [23]. Data were analyzed using R version 4.1.2 (Boston, Massachusetts, USA).

## 3 Results

### 3.1 Study population

**Fig. 1:**
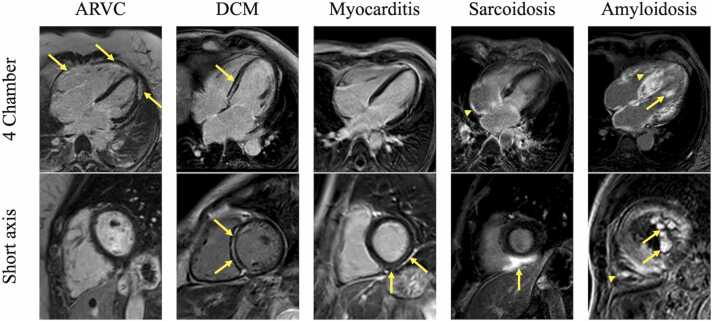
Examples of contrast-enhanced CMR images per diagnosis. Four-chamber (top row) and short-axis (bottom row) LGE images per diagnosis (columns). First column: patient with ARVC with a PKP2 gene mutation showing extensive LGE of the RV free wall and combined fibrofatty infiltration of the LV apical lateral wall. Second column: patient with DCM showing mid-myocardial septal LGE. Third column: patient who has recovered from myocarditis with mid-myocardial linear LGE in the inferior wall, extending to the inferoseptal and inferolateral wall. Fourth column: patient with fluorodeoxyglucose-positron emission tomography and biopsy confirmed cardiac sarcoidosis showing atrial LGE (arrowhead) and subepicardial LGE in the LV inferior wall extending into the inferoseptal wall and RV inferior wall (whale’s tail sign; arrow). Fifth column: patient with ATTR wild-type cardiac amyloidosis. The 4-chamber phase-sensitive inversion recovery sequence and the short-axis standard inversion recovery sequence show diffuse enhancement of both atria and ventricles, as well as enhancement of the papillary muscles (arrows) and the moderator band (arrowhead). *ARVC* arrhythmogenic right ventricular cardiomyopathy, *ATTR* amyloid transthyretin, *CMR* cardiovascular magnetic resonance, *DCM* dilated cardiomyopathy, *LGE* late gadolinium enhancement, *LV* left ventricular, *RV* right ventricular.

The study population consisted of 132 subjects clinically diagnosed with ARVC (n = 55), DCM (n = 25), (resolved) myocarditis (n = 13), cardiac sarcoidosis (n = 20), and cardiac amyloidosis (n = 19). Examples of contrast-enhanced CMR images per diagnosis are shown in Fig. 1. In total, 79 subjects (60%) were male, and mean age at the time of CMR was 47 ± 11 years. Baseline characteristics are presented in Table 1; familial history and genetic testing results from the ARVC and DCM groups can be found in Additional File: Table S1.

**Table 1:** Patient demographics and global parameters at cardiac magnetic resonance imaging.

	Overall (n = 132)	ARVC (n = 55)	Non-ARVC					p-value per diagnosis	p-value ARVC vs non-ARVC
			DCM (n = 25)	Myocarditis (n = 13)	Sarcoidosis (n = 20)	Amyloidosis (n = 19)	All non-ARVC (n = 77)		
Demographics									
Male sex (%)	79 (60)	28 (51)	14 (56)	10 (77)	13 (65)	14 (74)	51 (66)	0.258	0.112
Age (y)	47 ± 11	43 ± 19	44 ± 15	36 ± 13	50 ± 12	67 ± 9	50 ± 17	<0.001	0.044
Global CMR parameters									
LV CMR parameters									
EF (%)	52 (40–56)	54 (51–57)	25 (16–35)	54 (51–59)	52 (36–56)	47 (41–55)	42 (30–54)	<0.001	<0.001
EDV/BSA	100 (88–115)	97 (86–106)	163 (141–186)	96 (84–103)	96 (78–109)	100 (86–112)	105 (89–149)	<0.001	0.006
ESV/BSA	48 (39–69)	43 (38–52)	120 (90-157)	44 (37–47)	45 (36–61)	53 (37–72)	60 (41–92)	<0.001	<0.001
SV	92 ± 27	103 ± 22	73 ± 29	91 ± 30	90 ± 28	88 ± 19	84 ± 28	<0.001	<0.001
LGE (%)	98 (74)	28 (51)	18 (72)	13 (100)	20 (100)	19 (100)	70 (91)	<0.001	<0.001
RV-CMR parameters									
EF (%)	47 (39–54)	47 (40–51)	37 (23–47)	55 (53–61)	47 (43–53)	52 (45–59)	48 (39–54)	<0.001	0.449
EDV/BSA	101 ± 25	111 ± 22	105 ± 30	89 ± 27	92 ± 20	85 ± 18	94 ± 24	<0.001	<0.001
ESV/BSA	52 (42–69)	57 (46–75)	56 (52–100)	42 (31–51)	47 (40–56)	42 (33–51)	49 (39–58)	<0.001	0.113
SV	86 ± 27	100 ± 19	64 ± 32	90 ± 30	86 ± 27	84 ± 15	79 ± 29	<0.001	0.014
LGE (%)	66 (50)	21 (38)	4 (16)	9 (69)	16 (80)	16 (84)	45 (58)	<0.001	0.034

Patient and CMR characteristics. Data are numbers (%) of cases, means ± standard deviation, or medians (interquartile range). A p-value of <0.004 was considered statistically significant in this table after Bonferroni correction.

*ARVC* arrhythmogenic right ventricular cardiomyopathy*, BSA* body surface area*, CMR* cardiovascular magnetic resonance *, DCM* dilated cardiomyopathy*, EDV* end-diastolic volume*, EF* ejection fraction*, ESV* end-systolic volume*, LGE* late gadolinium enhancement*, LV* left ventricular*, RV* right ventricular*, SV* stroke volume*, y* years*.*

The included groups were comparable based on sex (p = 0.258), while age was significantly different across groups (p < 0.001): the youngest patients were observed in the myocarditis (36 ± 13 years) and ARVC (43 ± 19 years) groups, whereas amyloidosis patients (67 ± 9 years) were the oldest. Both RV and LV volumes and function were significantly different across groups (see Table 1): more specifically, RV volumes were significantly higher in ARVC patients (111 ± 22 mL/m^2^ ARVC vs 94 ± 24 mL/m^2^ non-ARVC, p < 0.001), whereas LV EF was significantly lower in non-ARVC patients (42% [30%−54%] non-ARVC vs 54% [51%−57%] ARVC, p < 0.001).

### 3.2 Presence and distribution of right ventricular LGE

**Fig. 2:**
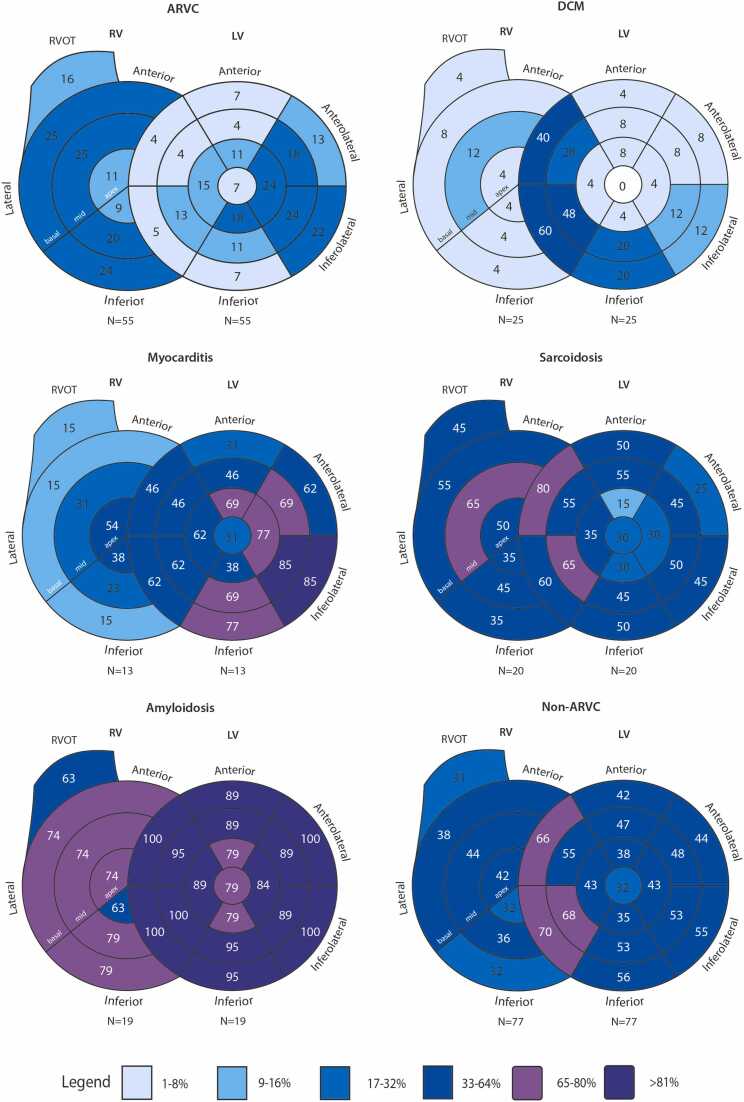
Distribution pattern of LGE per diagnosis. Overview of LGE distribution between diagnoses. Numbers per segment represent percentages of the respective diagnosis. *ARVC* arrhythmogenic right ventricular cardiomyopathy, *DCM* dilated cardiomyopathy, *LGE* late gadolinium enhancement, *LV* left ventricular, *RV* right ventricular, *RVOT* right ventricular outflow tract.

A total of 66 (50%) patients had RV-LGE. Patterns of RV-LGE per diagnosis are visually depicted in Fig. 2, while distribution of hyper-enhanced segments is compared in Table 2.

**Table 2:** Presence and distribution of right ventricular and miscellaneous late gadolinium enhancement between ARVC and its differentials.

	Overall (n = 132)	ARVC (n = 55)	Non-ARVC					p-value per diagnosis	p-value ARVC vs non-ARVC
			DCM (n = 25)	Myocarditis (n = 13)	Sarcoidosis (n = 20)	Amyloidosis (n = 19)	All non-ARVC (n = 77)		
Right ventricular segments									
RV-LGE present	66 (50.0)	21 (38.2)	4 (16.0)	9 (69.2)	16 (80.0)*	16 (84.2)*	45 (58.4)	<0.001	0.034
Basal LGE	48 (36.4)	16 (29.1)	2 (8.0)	3 (23.1)	11 (55.0)	16 (84.2)*	32 (41.6)	<0.001	0.199
Basal inferior	38 (28.8)	13 (23.6)	1 (4.0)	2 (15.4)	7 (35.0)	15 (78.9)*	25 (32.5)	<0.001	0.363
Basal lateral	43 (32.6)	14 (25.5)	2 (8.0)	2 (15.4)	11 (55.0)	14 (73.7)*	29 (37.7)	<0.001	0.198
Mid LGE	53 (40.2)	15 (27.3)	3 (12.0)	5 (38.5)	14 (70.0)*	16 (84.2)*	38 (41.6)	<0.001	0.018
Mid inferior	39 (29.5)	11 (20.0)	1 (4.0)	3 (23.1)	9 (45.0)	15 (78.9)*	28 (36.4)	<0.001	0.066
Mid lateral	48 (36.4)	14 (25.5)	3 (12.0)	4 (30.8)	13 (65.0)	14 (73.7)*	34 (44.2)	<0.001	0.044
Apical LGE	43 (32.6)	8 (14.5)	6 (18.8)	9 (69.2)*	11 (55.0)*	14 (73.7)*	35 (41.6)	<0.001	<0.001
Apical inferior	30 (22.7)	8 (14.5)	1 (4.0)	5 (38.5)	7 (35.0)	12 (63.2)*	25 (32.5)	<0.001	0.002
Apical lateral	38 (28.8)	6 (10.6)	1 (4.0)	7 (53.8)*	10 (50.0)*	14 (73.7)*	32 (41.6)	<0.001	<0.001
RV outflow tract	33 (25.0)	9 (16.4)	1 (4.0)	2 (15.4)	9 (45.0)	12 (63.2)*	24 (31.2)	<0.001	0.083
Median number of RV-LGE segments	1 (0–4)	0 (0–3)	0 (0–0)	1 (0–3)	3 (2–4)*	6 (3–6)*	1 (0–5)	<0.001	0.007
Miscellaneous segments									
Atrial LGE	26 (19.7)	1 (1.8)	2 (8.0)	1 (7.7)	4 (20.0)	18 (94.7)*	25 (32.5)	<0.001	<0.001
Valvular LGE	17 (12.9)	4 (7.3)	0 (0.0)	0 (0.0)	0 (0.0)	13 (68.4)*	13 (16.9)	<0.001	0.121
Papillary muscle LGE	23 (17.4)	0 (0.0)	0 (0.0)	1 (7.7)	7 (35.0)*	15 (78.9)*	23 (29.9)	<0.001	<0.001
Moderator band LGE	21 (15.9)	0 (0.0)	0 (0.0)	1 (7.7)	8 (40.0)*	12 (63.2)*	21 (27.3)	<0.001	<0.001
Whale’s Tail sign	13 (9.8)	0 (0.0)	1 (4.0)	1 (7.7)	11 (55.0)*	0 (0.0)	13 (16.9)	<0.001	0.004
LGE in any one of the above	41 (31.1)	4 (7.3)	2 (8.0)	3 (23.1)	14 (70.0)*	18 (94.7)*	37 (48.1)	<0.001	<0.001

Data are numbers (%) of cases, means ± standard deviation, or medians (interquartile range). A p-value <0.003 was considered statistically significant in this table after Bonferroni correction. Asterisk denotes statistically significant differences between the respective ARVC differential and ARVC.

*ARVC* arrhythmogenic right ventricular cardiomyopathy*, DCM* dilated cardiomyopathy*, LGE* late gadolinium enhancement*, RV* right ventricular.

Overall, 21 (38%) ARVC patients had RV-LGE. As shown in Table 2, RV-LGE in ARVC patients was most commonly observed in the basal segments (n = 16 [29%]; most often basal lateral wall n = 14 [26%]), followed by the mid-segments (n = 15 [27%]; most often mid-lateral wall n = 14 [26%]). RV-LGE was least often observed in the apical segments (n = 8 [15%]). The median number of hyper-enhanced RV segments in ARVC patients was 0 [0–3] per patient.

On average, non-ARVC patients had, although not statistically significant, more often RV-LGE as compared to ARVC patients (38% vs 58%, p = 0.034, significance threshold p < 0.003). As shown in Table 2, disease-specific differences were observed, where DCM patients had lower number of hyper-enhanced RV segments (median 0 [0] segments) and amyloidosis patients had widespread RV-LGE with a high number of hyper-enhanced RV segments (median 6 [3–6] segments).

### 3.3 Presence and distribution of left ventricular LGE

**Fig. 3:**
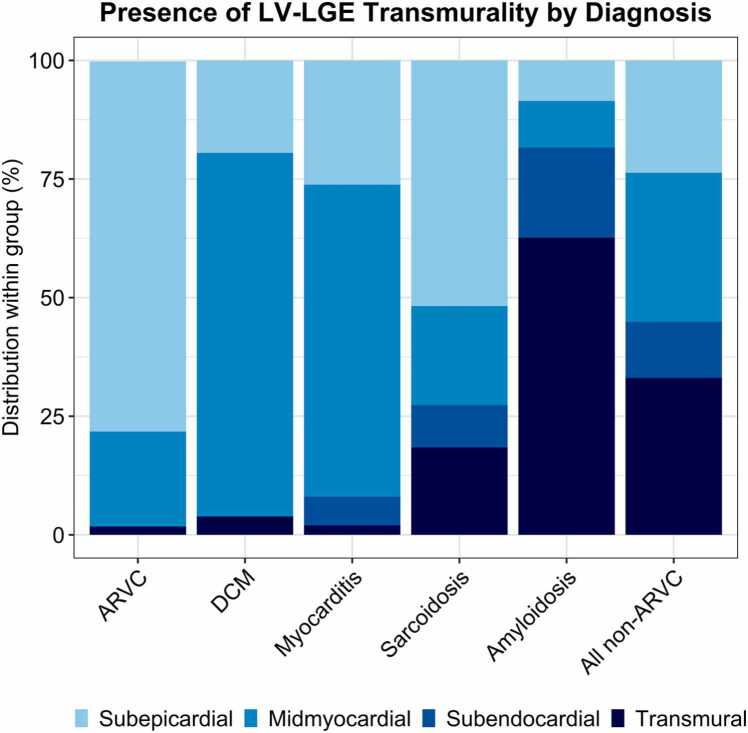
Transmurality of LV-LGE by diagnosis. Distribution of transmurality among diagnosis. All 98 patients who had LV-LGE are represented in this graph, stratified by diagnosis. Each diagnosis is scaled to 100% to show the proportion of transmurality in each group. *ARVC* arrhythmogenic right ventricular cardiomyopathy, *DCM* dilated cardiomyopathy, *LGE* late gadolinium enhancement, *LV* left ventricular.

A total of 98 (74%) patients had LV-LGE. Patterns of LV-LGE per diagnosis are visually depicted in Fig. 2. Distribution of hyper-enhanced LV segments per diagnosis is compared in Table 3 along with the transmurality of these segments in Fig. 3.

**Table 3:** Presence and distribution of left ventricular late gadolinium enhancement between ARVC and its differentials.

	Overall (n = 132)	ARVC (n = 55)	Non-ARVC					p-value per diagnosis	p-value ARVC vs non-ARVC
			DCM (n = 25)	Myocarditis (n = 13)	Sarcoidosis (n = 20)	Amyloidosis (n = 19)	All non-ARVC (n = 77)		
LV-LGE present	98 (74.2)	28 (50.9)	18 (72.0)	13 (100.0)*	20 (100.0)*	19 (100.0)*	70 (90.9)	<0.001	<0.001
Anterior	44 (33.3)	5 (9.1)	2 (8.0)	7 (53.8)*	13 (65.0)*	17 (89.5)*	39 (50.6)	<0.001	<0.001
Basal anterior	36 (27.6)	4 (7.3)	1 (4.0)	4 (30.8)	10 (50.0)*	17 (89.5)*	32 (41.6)	<0.001	<0.001
Mid anterior	38 (28.8)	2 (3.6)	2 (8.0)	6 (46.2)*	11 (55.0)*	17 (89.5)*	36 (46.8)	<0.001	<0.001
Anteroseptal	58 (43.9)	3 (5.5)	12 (48.0)*	6 (46.2)*	18 (90.0)*	19 (100.0)*	55 (71.4)	<0.001	<0.001
Basal anteroseptal	53 (40.2)	2 (3.6)	10 (40.0)*	6 (46.2)*	16 (80.0)*	19 (100.0)*	51 (66.2)	<0.001	<0.001
Mid anteroseptal	44 (33.3)	2 (3.6)	7 (28.0)	6 (46.2)*	11 (55.0)*	18 (94.7)*	42 (54.5)	<0.001	<0.001
Anterolateral	56 (42.4)	13 (23.6)	3 (12.0)	10 (76.9)*	11 (55.0)	19 (100.0)*	43 (55.8)	<0.001	<0.001
Basal anterolateral	41 (31.1)	7 (12.7)	2 (8.0)	8 (61.5)*	5 (25.0)	19 (100.0)*	34 (44.2)	<0.001	<0.001
Mid anterolateral	47 (35.6)	10 (18.2)	2 (8.0)	9 (69.2)*	9 (45.0)	17 (89.5)*	37 (48.1)	<0.001	<0.001
Inferior	56 (42.4)	7 (12.7)	6 (24.0)	11 (84.6)*	13 (65.0)*	19 (100.0)*	49 (63.6)	<0.001	<0.001
Basal inferior	47 (35.6)	4 (7.3)	5 (20.0)	10 (76.9)*	10 (50.0)*	18 (94.7)*	43 (55.8)	<0.001	<0.001
Mid inferior	47 (35.6)	6 (10.9)	5 (20.0)	9 (69.2)*	9 (45.0)*	18 (94.7)*	41 (53.2)	<0.001	<0.001
Inferoseptal	67 (50.8)	8 (14.5)	15 (60.0)*	9 (69.2)*	16 (80.0)*	19 (100.0)*	59 (76.6)	<0.001	<0.001
Basal inferoseptal	57 (43.2)	3 (5.5)	15 (60.0)*	8 (61.5)*	12 (60.0)*	19 (100.0)*	54 (70.1)	<0.001	<0.001
Mid inferoseptal	59 (44.7)	7 (12.7)	12 (48.0)*	8 (61.5)*	13 (65.0)*	19 (100.0)*	52 (67.5)	<0.001	<0.001
LGE inferolateral	65 (49.2)	18 (32.7)	3 (12.0)	12 (92.3)*	13 (65.0)	19 (100.0)*	47 (61.0)	<0.001	0.002
Basal inferolateral	54 (40.9)	12 (21.8)	3 (12.0)	11 (84.6)*	9 (45.0)	19 (100.0)*	42 (54.5)	<0.001	<0.001
Mid inferolateral	54 (40.9)	13 (23.6)	3 (12.0)	11 (84.6)*	10 (50.0)	17 (89.5)*	41 (53.2)	<0.001	0.001
Apex	59 (44.7)	16 (29.1)	2 (8.0)	12 (92.3)*	11 (55.0)	18 (94.7)*	43 (55.8)	<0.001	0.004
Apical anterior	35 (26.5)	6 (10.9)	2 (8.0)	9 (69.2)*	3 (15.0)	15 (78.9)*	29 (37.7)	<0.001	0.001
Apical septal	41 (31.1)	8 (14.5)	1 (4.0)	8 (61.5)*	7 (35.0)	17 (89.5)*	33 (42.9)	<0.001	0.001
Apical inferior	37 (28.0)	10 (18.2)	1 (4.0)	5 (38.5)	6 (30.0)	15 (78.9)*	27 (35.1)	<0.001	0.040
Apical lateral	46 (34.8)	13 (23.6)	1 (4.0)	10 (76.9)*	6 (30.0)	16 (84.2)*	33 (42.9)	<0.001	0.027
True apex	29 (22.0)	4 (7.3)	0 (0.0)	4 (30.8)	6 (30.0)	15 (78.9)*	25 (32.5)	<0.001	0.001
Median number of LV-LGE segments	4 (0–10)	0 (0–3)	2 (0–4)	10 (7–13)*	8 (5–10)*	17 (17–17)*	9 (3–13)	<0.001	<0.001

Data are numbers (%) of cases, means ± standard deviation, or medians (interquartile range). A p-value of <0.002 was considered statistically significant in this table after Bonferroni correction. Asterisk denotes statistically significant differences between respective ARVC differential and ARVC. Abbreviations as in text.

*ARVC* arrhythmogenic right ventricular cardiomyopathy*, DCM* dilated cardiomyopathy*, LGE* late gadolinium enhancement*, LV* left ventricular.

Overall, 28 (51%) ARVC patients had LV-LGE. As shown in Table 3, LV-LGE in ARVC patients was most commonly observed in the inferolateral wall (n = 18 [33%]) followed by the apex (n = 16 [29%]). LV-LGE was least often observed in the anterior or anteroseptal wall (n = 5 [9%], n = 3 [6%], respectively). The median number of hyper-enhanced LV segments in ARVC patients was 0 [0–3] per patient. Of note, the majority (n = 89/113 segments [79%]) of LV-LGE in ARVC patients was observed in a subepicardial pattern (Fig. 3).

On average, non-ARVC patients were more likely than ARVC patients to have LV-LGE (91% vs 51%, p < 0.001). Regional differences were observed, where DCM and sarcoidosis patients most often had LGE in the septum (48% and 90% for anteroseptum; 60% and 80% for inferoseptum, respectively); myocarditis patients typically had LGE in the lateral wall (92% inferolateral and 77% anterolateral wall); and amyloidosis patients had widespread LGE in almost all segments (Table 3). Consequently, non-ARVC patients had more extensive LV-LGE with a higher median number of hyper-enhanced LV segments (median 9 [3–13] segments, p < 0.001 compared to ARVC). In addition, Fig. 3 shows that LV-LGE was significantly more often mid-myocardial in DCM and myocarditis patients (n = 59/77 [77%] (p < 0.001) and n = 98/149 [66%] (p < 0.001) segments, respectively, compared to ARVC), whereas it was typically transmural in amyloidosis patients (n = 198/316 [63%], p < 0.001 in comparison to ARVC).

### 3.4 Other LGE patterns

We also evaluated the presence of Whale’s Tail sign and atrial, valvular, papillary muscle, and moderator band LGE. As shown in Table 2, 7% (n = 4/55) of the ARVC patients had LGE in any of these locations, compared to 48% (n = 37/77) of non-ARVC patients (p < 0.001). This was largely driven by the frequent observation of atrial, valvular, and papillary muscle LGE in amyloidosis patients (n = 18 [95%] atrial, n = 13 [68%] valvular, n = 15 [79%] papillary muscle, and n = 12 [63%] moderator band LGE) and in sarcoidosis patients (n = 4 [20%] atrial, n = 7 [35%] papillary muscle, n = 8 [40%] moderator band LGE and n = 11 [55%] presence of the Whale’s Tail sign).

### 3.5 LGE for ARVC diagnosis

**Fig. 4:**
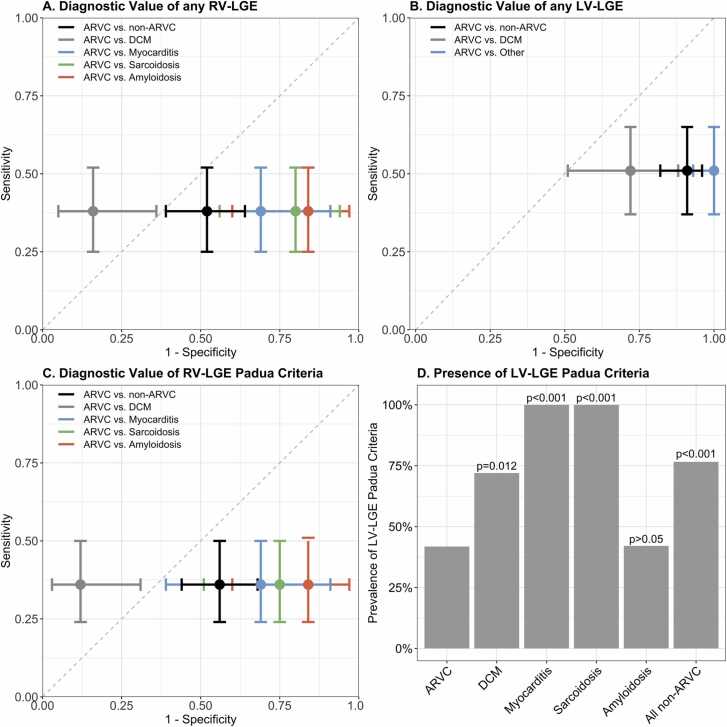
Prevalence and diagnostic value of RV-LGE and LV-LGE per Padua criteria for ARVC and its differentials. Sensitivity (y-axis) and 1-specificity (x-axis) of (A) any RV-LGE and (B) any LV-LGE to differentiate between ARVC and non-ARVC (black), DCM (gray), myocarditis (blue), sarcoidosis (green), and amyloidosis (orange). (C) Sensitivity (y-axis) and 1-specificity (x-axis) of RV-LGE per Padua criteria to differentiate between ARVC and non-ARVC (black), DCM (gray), and a composite of myocarditis, sarcoidosis, and amyloidosis (light blue). Myocarditis, sarcoidosis, and amyloidosis all had a specificity of 0% and a sensitivity of 34% and were therefore visualized as a composite measure. The gray dashed line in A-C indicates the line of no discrimination. Since LGE was described as a binary variable, dots and error bars (denoting 95% confidence intervals) are shown, instead of a line graph. A dot on the left-upper side of the line of no discrimination favors a positive association of the variable with ARVC diagnosis, whereas a dot on the right-lower side of the line of no discrimination favors a negative association of the variable with ARVC diagnosis. (D) Presence of LV-LGE per Padua criteria (y-axis) stratified by diagnosis (x-axis). Statistical difference of LV-LGE presence is denoted on top of every bar. *ARVC* arrhythmogenic right ventricular cardiomyopathy, *DCM* dilated cardiomyopathy, *LGE* late gadolinium enhancement, *LV* left ventricular, *RV* right ventricular.

Next, we analyzed the diagnostic value of (1) “any RV-LGE”; (2) “any LV-LGE”; (3) “RV-LGE per Padua criteria”; and (4) “LV-LGE per Padua criteria” to distinguish ARVC from its differentials. Results are shown in Fig. 4 and described below. The intra- and interobserver variabilities are tabulated in Additional file 1: Table S2 and were good.

#### 3.5.1 “Any” right and left ventricular LGE

Fig. 4A visualizes the diagnostic value of any RV-LGE between ARVC vs non-ARVC and each differential separately. As can be appreciated, any RV-LGE yielded poor diagnostic value for ARVC vs non-ARVC (accuracy 40%, sensitivity 38%, specificity 42%, positive predictive value 32%, and negative predictive value 48%). Sub-analysis for each differential separately yielded a good discrimination between ARVC and DCM (specificity 84%), whereas it was poor for all other diagnoses (specificity ≤31%). Similarly, as shown in Fig. 4B, presence of any LV-LGE yielded poor diagnostic value between ARVC and all differentials with particularly low specificity (accuracy ≤44%, sensitivity 51%, specificity ≤28%, positive predictive value ≤61%, negative predictive value ≤21%). This indicates that LV-LGE is more likely to occur in those with a non-ARVC diagnosis.

#### 3.5.2 Right and left ventricular LGE fulfillment per Padua criteria

We subsequently evaluated the diagnostic value of LGE per Padua criteria to distinguish ARVC from its differentials. As specified in Section 2, we report both prevalence per diagnosis and conventional diagnostic metrics for RV-LGE per Padua criteria, whereas we report only prevalence per diagnosis for LV-LGE per Padua criteria.

The study population is stratified by LGE per Padua criteria in Additional File: Table S3. In short, fulfillment of both LV-LGE and RV-LGE, only fulfillment of LV-LGE per Padua criteria, only fulfillment of RV-LGE per Padua criteria, and neither fulfillment of LV- nor RV-LGE per Padua Criteria was present in 43 (33%), 39 (30%), 20 (15%), 30 (23%) patients, respectively. Patients did not statistically differ in age, sex, or global CMR parameters.

Additional File: Fig. S3 shows the prevalence of RV-LGE per Padua criteria for ARVC and its differentials. As shown, RV-LGE per Padua criteria was present in 20 [36%] ARVC patients compared to 43 [56%] non-ARVC patients (p = 0.027). This culminated in an accuracy of 41% including a sensitivity of 36%, specificity of 44%, positive predictive value of 32%, and negative predictive value of 49% for ARVC diagnosis (Fig. 4C). Sub-analysis for each differential showed a good differentiation between ARVC and DCM (specificity 88%), whereas it was poor for all other diagnoses (specificity ≤31%) (Fig. 4C).

LV-LGE per Padua criteria were present in 23 [42%] ARVC patients compared to 59 [77%] non-ARVC patients (p < 0.001). As can be appreciated in Fig. 4D, LV-LGE per Padua criteria were particularly likely to be observed in myocarditis and sarcoidosis patients (100% in each of these differentials, p < 0.001 compared to ARVC), but also observed in three quarters of DCM patients (n = 18/25 [72%], p = 0.012 compared to ARVC). LV-LGE per Padua criteria were comparable between ARVC and amyloidosis patients (n = 8/19 [42%], p = 0.983 compared to ARVC).

#### 3.5.3 Sensitivity analysis

In total, 40 (73%) of ARVC patients carried a (likely) pathogenic variant (i.e. “genetically proven ARVC” group) in which we subsequently assessed the diagnostic value of LGE as a sensitivity analysis. The pattern of LGE in the ARVC patients carrying a (likely) pathogenic variant is provided in Additional File: Fig. S4. As visualized in Additional File: Fig. S5, any LV-LGE, any RV-LGE, RV-LGE per Padua criteria, and presence of LV-LGE per Padua criteria yielded similar results in the “genetically proven ARVC” group as compared to the overall ARVC group.

### 3.6 Pattern analysis

Last, we set out to determine if a data-driven approach could optimize the diagnostic value of LGE in the context of ARVC evaluation.

**Fig. 5:**
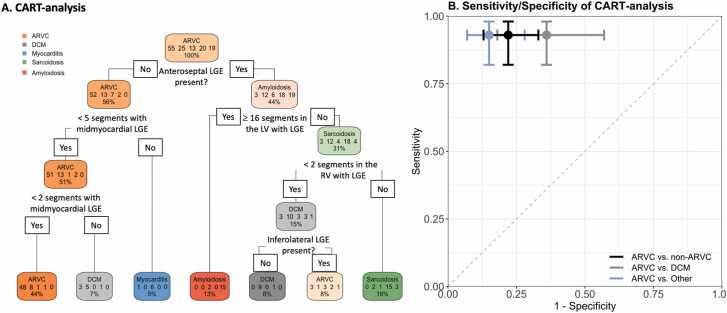
CART analysis. (A) CART analysis of our study population. In each box, the predicted diagnosis, the numbers of observed diagnosis, and the fraction of the total study population are denoted from top to bottom, respectively. The numbers in the observed diagnosis indicate ARVC, DCM, myocarditis, sarcoidosis, and amyloidosis, respectively. (B) Sensitivity (y-axis) and 1-specificity (x-axis) of the CART analysis to differentiate between ARVC and non-ARVC (black), DCM (gray), and a composite of myocarditis, sarcoidosis, and amyloidosis (light blue). Myocarditis, sarcoidosis, and amyloidosis all had a specificity of 100% and a sensitivity of 94% and were therefore visualized as a composite measure. The gray dashed line indicates the line of no discrimination. Since LGE was described as a binary variable, dots and error bars (denoting 95% confidence intervals) are shown, instead of a line graph. A dot on the left-upper side of the line of no discrimination favors a positive association of CART fulfillment with ARVC diagnosis, whereas a dot on the right-lower side of the line of no discrimination favors a negative association of CART fulfillment with ARVC diagnosis. *ARVC* arrhythmogenic right ventricular cardiomyopathy, *CART* classification and regression tree, *DCM* dilated cardiomyopathy, *LGE* late gadolinium enhancement, *LV* left ventricular, *RV* right ventricular.

Fig. 5A shows the results of our CART analysis. As shown in this figure, a combination of “absence of anteroseptal LV-LGE,” “absence of extensive (≥5 segments) mid-myocardial LV-LGE,” “absence of moderate (≥2 segments) mid-myocardial LV-LGE,” or “presence of anteroseptal LV-LGE,” “absence of extensive (≥16 segments) LV-LGE,” “absence of moderate (≥2 segments) RV-LGE,” and “presence of inferolateral LGE” discriminated between ARVC differentials. As can be appreciated in Fig. 5B, this yielded good discriminatory potential between ARVC and non-ARVC (accuracy 84%, sensitivity 93%, specificity 78%, positive predictive value 75%, and negative predictive value 94%). In addition, the CART analysis showed discriminatory potential between ARVC and each differential separately. Specifically, the discrimination between ARVC and myocarditis (by “absence of extensive [≥5 segments] mid-myocardial LV-LGE”), amyloidosis (by “absence of extensive [≥16 segments] LV-LGE”), and sarcoidosis (by “absence of moderate [≥2 segments] RV-LGE”) was good (specificity 85%), while it was moderate between ARVC and DCM (specificity 64%).

## 4 Discussion

Objective data testing the diagnostic value of LGE (and in particular its specificity, i.e. the potential to discriminate between ARVC and its differentials) within the spectrum of arrhythmogenic cardiomyopathies is lacking. This study aimed to fill this knowledge gap by systematically evaluating LGE in a cohort of extensively phenotyped individuals who were evaluated with a dedicated RV-CMR protocol. This manuscript has three main findings, that may be summarized as follows:

− RV-LGE is observed in approximately one-third of ARVC patients, typically in the RV inlet region (i.e. basal RV wall). LV-LGE is observed in half of ARVC patients, most frequently in the LV inferolateral region in a subepicardial pattern.
− At the same time, LGE is consistently present in ARVC differentials. As such, while LGE may indeed be helpful in determining the presence of focal fibrosis, the crude inclusion of its presence without any reference to the patterns of distribution may result in a large number of false positive diagnoses of ARVC.
− When using a data-driven approach, a combination of LGE location (i.e. LV anteroseptal LGE), transmurality (i.e. LV mid-myocardial LGE), and extent (i.e. number of segments with LV and RV-LGE) is useful for diagnostic classification. While this approach needs to be externally validated, it serves as a proof of concept that LGE patterns may enable more precise identification of differentials within the spectrum of arrhythmogenic cardiomyopathies.

### 4.1 Diagnostic paradigm shift within the spectrum of arrhythmogenic cardiomyopathies

There is no standard of reference test that is pathognomonic for ARVC. Therefore, a complex set of diagnostic TFC that integrates different aspects of clinical evaluation was first proposed in 1994 [24], and subsequently revised in 2010 [7] to allow for more sensitivity for early disease [25]. In recent years, however, a critical appraisal of the 2010 TFC by an international panel of experts revealed the need for improvements in the criteria and formulated the goal to include a broader spectrum of phenotypes (e.g. LV phenotypes) as well as non-invasive methods of tissue characterization (e.g. LGE criteria) [26].

Around the same time, a consensus statement published by the HRS provided a new (albeit separate) definition of ACM as an “arrhythmogenic heart muscle disorder not explained by ischemic, hypertensive, or valvular heart disease” [6]. This HRS umbrella definition therefore includes, apart from ARVC, a broad spectrum of systemic (e.g. sarcoidosis, amyloidosis), inflammatory/infectious (e.g. myocarditis, Chagas disease), and (left-dominant) genetic (e.g. Lamin A/C [*LMNA*]*,* Filamin C [*FLNC*]) heart diseases. This HRS definition significantly differs from the more specific definition that is typically used in clinical and experimental settings, in which ACM is regarded as a genetic cardiomyopathy affecting the RV, LV, or both, and predisposes the patient to life-threatening arrhythmias irrespective of the presence of global and/or regional dysfunction. Regardless, it provides a theoretical framework for ACM management and an overview of ARVC differentials within the broad ACM spectrum, which we exploit for the present study.

### 4.2 Identification of fibrofatty infiltration by LGE in ARVC

The first important finding of our study is that both RV-LGE and LV-LGE are present in a significant proportion of ARVC patients. Since the first report on RV-LGE in ARVC patients in 2005 [5], presence of RV-LGE has been consistently reported in ARVC in up to 88% of affected individuals [27]. It is important to recognize that several studies have shown that structural abnormalities, such as RV-LGE, are usually preceded by electrical (i.e. ECG, Holter monitoring) abnormalities in ARVC [4], [28]. As such, our low prevalence of RV-LGE may be explained by the fact that we diagnosed ARVC patients in a relatively early stage of disease as compared to older reports with higher prevalence of RV-LGE [5], [27], that ARVC patients over the years are diagnosed at an earlier phase of disease as exemplified by our relatively low prevalence of RV-LGE. Of note, RV-LGE in our cohort predominantly affected the basal RV (i.e. basal lateral/inferior wall and RV outflow tract) and spared the RV apex. This may be an important finding for clinical practice, as the RV apex is thin and often difficult to assess, leading to a frequent cause of misdiagnosis in ARVC.

In addition, half of our ARVC patients had LV-LGE. More specifically, LV-LGE in ARVC patients typically affected the subepicardial inferolateral LV with preserved LV function. Of note, previous studies suggested that LV involvement is genotype-specific, and more often observed in non-*PKP2* pathogenic variant carriers [29]. However, even in our cohort with predominantly *PKP2* pathogenic variant carriers, LV-LGE in the inferolateral wall was more prevalent than RV apical involvement, leading us to previously coin the “displacement” of the Triangle of Dysplasia that traditionally described abnormalities in ARVC [30]. Recognition of this pattern of involvement may help the clinician to correctly establish an ARVC diagnosis.

### 4.3 Diagnostic value of LGE for diagnosing ARVC

The second important finding of our study is that LGE is often present in ARVC differentials. Specifically, RV-LGE and LV-LGE in our study were more often present in non-ARVC as compared to ARVC patients. As such, the presence of “any RV-LGE” (accuracy 40%) and “any LV-LGE” (accuracy 27%) provided poor discrimination between ARVC and its differentials.

As with all diagnostic criteria, implementing LGE in the diagnostic criteria for ARVC will result in a “sensitivity/specificity trade-off.” We would argue that in the case of distinguishing ARVC from its differentials, favoring higher specificity would be preferable: misdiagnosing a myocarditis, sarcoidosis, or amyloidosis patient with ARVC could have large therapeutic and prognostic implications (also for the individual’s family members). Of note, we acknowledge that amyloidosis has very different clinical and CMR imaging features as compared to other diseases, however, the diagnostic value (i.e. accuracy) of RV/LV-LGE remained poor between ARVC and other differentials (Fig. 4). As such, given the observed low specificity that was observed in our study, we believe that the use of “any RV-LGE” and “any LV-LGE” for ARVC evaluation is not advisable, since it may lead to a large number of false positive diagnoses.

### 4.4 Diagnostic value of RV-LGE per Padua criteria for diagnosing ARVC

In response to the above-mentioned paradigm shift (i.e. moving from ARVC as a unique RV-predominant disease, to ARVC as a genetically distinct subtype of ACM), the Padua criteria were developed to incorporate both LV phenotypes (i.e. arrhythmogenic left ventricular cardiomyopathy [ALVC]) and non-invasive detection of fibrofatty replacement by LGE [8]. This study evaluated the LGE criterion of the Padua criteria framework for both the RV and LV.

The Padua Criteria mandate the presence of morphological and/or structural alterations (i.e. RV-LGE) to classify the patient as ARVC, which was implemented to improve diagnostic specificity. However, the widespread presence of this finding in ARVC differentials and its consequently low specificity may cause gene-elusive DCM, myocarditis, sarcoidosis, and amyloidosis patients to be potentially diagnosed with ARVC, with LGE as the “mis-diagnostic point of entry” of these patients. Given the extensive differences in risk stratification and management (i.e. immunosuppression, exercise detraining, etc) between a final diagnosis of ARVC and sarcoidosis, it is of paramount importance to limit the diagnostic overlap. A potential solution for this problem would be to increase the pre-test probability for ARVC diagnosis: e.g. by only enabling the RV-LGE criterion to be fulfilled in patients carrying an ARVC-associated (likely) pathogenic variant. This choice would mirror the one the authors of the Padua criteria made for the LV-LGE criterion to prevent misdiagnosis, as a more widespread, non-genetically based acceptance of LV-LGE as a diagnostic criterion would have large prognostic and therapeutic implications. While these limitations are important to keep in mind, the Padua criteria are an important initial step to shift the diagnostic paradigm toward recognizing left-dominant (“ALVC”) phenotypes and using non-invasive tissue characterization as a diagnostic criterion.

### 4.5 Data-driven LGE criteria for diagnosing ARVC

Our study also explored the possibility of creating more precise diagnostic LGE criteria for ARVC. Using our CART analysis as a proof of concept, we showed that a data-driven approach integrating all distinct LGE parameters may distinguish ARVC from its differentials with strong discriminatory potential. Of note, three distinct patterns of LGE can be appreciated when combining the CART analysis with visual data inspection: (1) a predominant RV-LGE distribution with some LV involvement, almost exclusively subepicardial in the inferolateral wall (i.e. ARVC); (2) a predominantly mid-myocardial LV-LGE distribution with some RV involvement (i.e. DCM and myocarditis); and (3) an equal LV and RV-LGE distribution (i.e. sarcoidosis and amyloidosis). Moreover, atrial, valvular papillary muscle, and moderator band LGE are almost solely present in patients with amyloidosis. Furthermore, the “Whale’s Tail” was highly specific for sarcoidosis, as it was only observed in sarcoidosis patients. Although previous studies show conflicting findings [30], [31], [32], we found a high prevalence of RV-LGE in myocarditis patients, many of whom had normal biventricular function. Recognition of these patterns may be helpful to clinicians when evaluating a patient for ARVC or its differentials and may limit possibly harmful misdiagnoses in patients who are evaluated for this rare, but potentially lethal disease.

### 4.6 Limitations and future perspectives on non-invasive tissue characterization for ARVC evaluation

As with any other study evaluating LGE, its detection in the RV is hampered by the thin RV wall, which may be even more pronounced in ARVC subjects. In addition, distinguishing fat from fibrosis by LGE may be challenging. We are aware of recent techniques that may provide more objective quantification of fibrosis in the myocardium. Specifically, a recent study showed that native T1 mapping differentiates patients with overt ARVC from at-risk relatives and controls [33], which indicates the potential value of new CMR techniques for ARVC diagnosis. Future studies should also evaluate their role in ARVC evaluation. Second, we did not include a dedicated “ALVC” group, which limited our ability to assess LGE as a diagnostic tool for these left-dominant ACM phenotypes. However, this limitation was insurmountable, given the lack of a widely accepted gold standard for ALVC. Next, while our study used a *phenotype*-driven comparison of LGE parameters, one could argue that the time has come to determine *genotype*-specific patterns of LGE involvement. Although we would be strong proponents of such an approach, we believe our current analyses remain important since (1) not all ARVC differentials have a genetic cause; (2) not all clinicians have access to genetic testing; and (3) widely accepted gene-specific management recommendations are currently lacking. Last, our findings regarding the RV-LGE per Padua criteria as well as the CART analysis warrant external validation. Of note, while we believe that our study convincingly shows that caution should be exercised when using LGE for diagnostic purposes, it may be a very useful parameter prognostically [34]. As such, future studies should scrutinize the prognostic value of LGE in predicting ventricular arrhythmias in ARVC patients.

## 5 Conclusion

This study evaluated the diagnostic value of LGE to distinguish ARVC from its differentials. We showed that RV-LGE is present in one-third of ARVC subjects, typically in the RV inlet region, and LV-LGE is present in half of ARVC patients, most commonly in the subepicardial LV inferolateral region. In addition, RV-LGE and/or LV-LGE is frequently present in ARVC differentials, resulting in a high false positive rate when including the sole presence of RV-LGE and/or LV-LGE as a diagnostic criterion (as exemplified by the Padua criteria). Pattern recognition may create more specific LGE criteria to differentiate ARVC from its differentials. Importantly, as with all diagnostic criteria, external validation is warranted before utilization of the proposed diagnostic flowchart.

## Funding

We acknowledge the support from the Netherlands Cardiovascular Research Initiative, an initiative with the support of the Netherlands Heart Foundation, grant nos.: CVON2015-12 eDETECT, 2020B005 Double Dose, and 2018-30 Predict 2. The Netherlands ACM Registry is supported by the 10.13039/501100014470Netherlands Heart Institute (project 06901). A.S.J.M.t.R. is supported by the ZonMW Off Road Grant 2021 and ERC HORIZON IMPACT (#101115536).

## Author contributions

**J. Peter van Tintelen:** Writing – review and editing, Supervision, Funding acquisition, Data curation, Conceptualization. **Marco Guglielmo:** Writing – review and editing, Supervision, Data curation. **Steven A. Muller:** Writing – review and editing, Writing – original draft, Visualization, Methodology, Investigation, Formal analysis, Data curation. **Birgitta K. Velthuis:** Writing – review and editing, Data curation, Conceptualization. **Alessio Gasperetti:** Writing – review and editing, Writing – original draft, Conceptualization. **Anneline S.J.M. te Riele:** Writing – review and editing, Writing – original draft, Supervision, Methodology, Data curation, Conceptualization. **Mimount Bourfiss:** Writing – review and editing, Data curation, Conceptualization. **Marish I.F.J. Oerlemans:** Writing – review and editing, Data curation, Conceptualization. **Maarten J. Cramer:** Writing – review and editing, Data curation, Conceptualization. **Stefan L. Zimmerman:** Writing – review and editing, Conceptualization. **Dennis Dooijes:** Writing – review and editing, Conceptualization. **Hanke Schalkx:** Writing – review and editing, Data curation, Conceptualization. **Pim van der Harst:** Writing – review and editing, Conceptualization. **Cynthia A. James:** Writing – review and editing, Conceptualization. **Lian Y. Rekker:** Writing – original draft, Visualization, Methodology, Investigation, Formal analysis, Data curation.

## Ethics approval and consent

The study protocol was exempt from the Medical Research Involving Human Subjects Act (WMO), as determined by the medical ethical committee at our hospital (protocol number 18-336/C).

## Consent for publication

Not applicable.

## Declaration of competing interests

The authors declare the following financial interests/personal relationships which may be considered as potential competing interests. A.G. has served as part of the advisory board of LEXEO Therapeutics for unrelated work. The remaining authors have no conflict of interest.

## Data Availability

The data underlying this article cannot be shared publicly due to the privacy of individuals who participated in the study. The data will be shared on reasonable request to the corresponding author.
