# Supplementary material for: Diagnostic value of late gadolinium enhancement at cardiovascular magnetic resonance to distinguish arrhythmogenic right ventricular cardiomyopathy from differentials
Source: J Cardiovasc Magn Reson. 2024 Jul 8;26(2):101059. doi: 10.1016/j.jocmr.2024.101059 (PMC11327940; doi:10.1016/j.jocmr.2024.101059)
Supplement: Supplementary file 1 — Supplementary material. [file mmc1.docx]

**ADDITIONAL FILE 1**

**Diagnostic Value of Late Gadolinium Enhancement at Cardiac Magnetic Resonance to distinguish Arrhythmogenic Right Ventricular Cardiomyopathy from Differentials**

Content

[Supplementary Tables 2](#_Toc167472265)

[Table S1. Genetic characteristics of ARVC and DCM groups 2](#_Toc167472266)

[Table S2. Intra- and interobserver variability 5](#_Toc167472267)

[Table S3. Study population stratified by LGE per Padua-criteria 6](#_Toc167472268)

[Supplementary Figures 8](#_Toc167472269)

[Figure S1. Ventricular segmentation 8](#_Toc167472270)

[Figure S2. Whale’s Tail sign in a patient with sarcoidosis 9](#_Toc167472271)

[Figure S3. Presence of RV-LGE per Padua Criteria 10](#_Toc167472272)

[Figure S4. Distribution pattern of LGE for “genetically proven ARVC”-group 11](#_Toc167472273)

[Figure S5. Prevalence and diagnostic value of RV-LGE and LV-LGE per Padua criteria for “genetically proven ARVC”-group and its differentials. 12](#_Toc167472274)

# **Supplementary Tables**

## **Table S1.** Genetic characteristics of ARVC and DCM groups

| **Patient** | **Family history** | **Gene** | **nucleotide** | **Amino acid** | **class** |
| --- | --- | --- | --- | --- | --- |
| ARVC-1 | Second-degree relative (aunt) with confirmed ARVC | *PLN* | c.40_42delAGA | p.(Arg14del) | Pathogenic |
| ARVC-2 | Second-degree relative (grandfather) with confirmed ARVC | *PKP2* | c.1211dupT | p.(Leu404fs) | Pathogenic |
| ARVC-3 | No family history of ARVC | *PKP2* | c.2489+4A>C | p.(? ) | Pathogenic |
| ARVC-4 | No family history of ARVC | *PLN* |  | Duplication of PLN gene | Pathogenic |
| ARVC-5 | First-degree relative (brother) with confirmed ARVC. Second-degree relative (niece, also PKP2 carrier) with SCD at age 27. | *PKP2* | c.917_918del | p.(Pro306fs) | Pathogenic |
| ARVC-6 | No family history of ARVC | gene-elusive |  |  |  |
| ARVC-7 | First-degree relative (father) and second-degree relatives (2 uncles (fathers' side)) with confirmed ARVC. | *PKP2* | c.235C>T | p.(Arg79*) | Pathogenic |
| ARVC-8 | Second-degree relative (uncle) with confirmed ARVC. | *DSG2* | c.137G>A | p.(Arg46Gln) | Pathogenic |
| ARVC-9 | Second-degree relative (nephew) with confirmed ARVC | *PKP2* | c.1848C>A | p.(Tyr616*) | Pathogenic |
| ARVC-10 | First-degree relative (father) with confirmed ARVC. | *PKP2* | c.2203C>T | p.(Arg735*) | Pathogenic |
| ARVC-11 | First-degree relative (father) with SCD at age 44. Second-degree relatives (uncle, aunt, (fathers' side)) with confirmed ARVC. | *PKP2* | c.235C>T | p.(Arg79*) | Pathogenic |
| ARVC-12 | First-degree relatives (sister and brother) with confirmed ARVC. Second degree relative (niece) confirmed ARVC and second-degree relative (nephew) SCD at age 17, subsequently confirmed ARVC by pathologist. | *PKP2* | c.397C>T | p.(Gln133*) | Pathogenic |
| ARVC-13 | First-degree relative (father) and second-degree relatives (2 aunts (fathers' side)) with confirmed ARFD; third degree relative (cousin) with SCD at age 17 and subsequently confirmed ARVC by pathologist. | *PKP2* | c.397C>T | p.(Gln133*) | Pathogenic |
| ARVC-14 | First-degree relative (father) and second-degree relative (uncle (fathers' side)) with confirmed ARVC. | *PKP2* | c.2544G>A | p.(Trp848*) | Pathogenic |
| ARVC-15 | First-degree relative (father) with confirmed ARVC. | *PKP2* | c.1211dup | p.(Val406fs) | pathogenic |
| ARVC-16 | First-degree relative (mother) with confirmed ARVC. | *PKP2* | c.2146-1G>C | p.(?) | Pathogenic |
| ARVC-17 | No family history of ARVC | *PKP2* | c.2014-1G>C | p.(?) | Pathogenic |
| ARVC-18 | First-degree relative (father) with confirmed ARVC. | *PKP2* | c.1-?_1170+?del (deletion exon 1-4) | p.(?) | Pathogenic |
| ARVC-19 | Second-degree relatives (uncle and aunt) with confirmed ARVC; third-degree relative (2 cousins): 1 had an SCD, and both were confirmed ARVC. | *PKP2* | c.1-?_2646+?del (deletion exon 1-14) | p.(?) | Pathogenic |
| ARVC-20 | First-degree relative (mother) with confirmed ARVC. | *PKP2* | c.1369_1372delCAAA | p.(Gln457*) | Pathogenic |
| ARVC-21 | First-degree relative (brother) with confirmed ARVC. Second-degree relative (uncle) with SCD at age 55. | *PKP2* | c.1369delCAAA | p.(Gln457fs) | Pathogenic |
| ARVC-22 | No family history of ARVC | *PKP2* | c.2146-1G>C | p.(?) | Pathogenic |
| ARVC-23 | First-degree relative (father) with confirmed ARVC. | *PKP2* | c.235C>T | p.(Arg79*) | Pathogenic |
| ARVC-24 | First-degree relative (2 daughters) withconfirmed ARVC. | *PKP2* | c.1211dup | p.(Val406fs) | Pathogenic |
| ARVC-25 | First-degree relative (father) with confirmed ARVC. Second-degree relative (uncle (fathers' side)) with SCD age 65. | *PKP2* | c.2146-1G>C | p.(?) | Pathogenic |
| ARVC-26 | First-degree relative (brother) with confirmed ARVC. | *PKP2* | c.55C>T | p.(Gln19*) | Pathogenic |
| ARVC-27 | Second-degree relative (grandfather) with SCD at age 35. | *PKP2* | c.1211dup | p.(Val406fs) | Pathogenic |
| ARVC-28 | First-degree relatives (brother and 2 sisters) with confirmed ARVC. | *PKP2* | c.1844C>T | p.(Ser615Phe) | Likely pathogenic |
| ARVC-29 | First-degree relative (father) and third-degree (niece) with confirmed ARVC | *PKP2* | c.397C>T | p.(Gln133*) | Pathogenic |
| ARVC-30 | First-degree relative (father) SCD at 46. | *PLN* | c.40_42delAGA | p.(Arg14del) | Pathogenic |
| ARVC-31 | First-degree relative (father) with confirmed ARVC. | *PKP2* | c.(?_-1612)_(1170+1_1171-1) del | p.(?) | Pathogenic |
| ARVC-32 | No family history of ARVC | *PKP2* | c.235C>T | p.(Arg79*) | Pathogenic |
| ARVC-33 | No family history of ARVC | *PLN* | c.40_42delAGA | p.(Arg14del) | Pathogenic |
| ARVC-34 | First-degree relative (brother) with SCD at age 28 | *PLN* | c.40_42delAGA | p.(Arg14del) | Pathogenic |
| ARVC-35 | No family history of ARVC | *PLN* | c.40_42delAGA | p.(Arg14del) | Pathogenic |
| ARVC-36 | First-degree relative (sister) with confirmed ARVC | *PLN* | c.40_42delAGA | p.(Arg14del) | Pathogenic |
| ARVC-37 | First-degree relative (father) and 2 second-degree relative (both aunts (fathers' side)) with confirmed ARVC. | *PLN* | c.40_42delAGA | p.(Arg14del) | Pathogenic |
| ARVC-38 | First-degree relatives (brother and sister) and second-degree relative (niece)) with confirmed ARVC. | *PLN* | c.40_42delAGA | p.(Arg14del) | Pathogenic |
| ARVC-39 | First-degree relatives (brother and sister) and second-degree relative (niece)) with confirmed ARVC. | *PLN* | c.40_42delAGA | p.(Arg14del) | Pathogenic |
| ARVC-40 | No family history of ARVC | *DSP* | c.3337C>T | p.(Arg1113*) | Pathogenic |
| ARVC-41 | No family history of ARVC | *DSP* | c.1048T>C | p.(Tyr350His) | Likely Pathogenic |
| ARVC-42 | No family history of ARVC | gene-elusive |  |  |  |
| ARVC-43 | No family history of ARVC | gene-elusive |  |  |  |
| ARVC-44 | No family history of ARVC | gene-elusive |  |  |  |
| ARVC-45 | No family history of ARVC | gene-elusive |  |  |  |
| ARVC-46 | No family history of ARVC | gene-elusive |  |  |  |
| ARVC-47 | No family history of ARVC | gene-elusive |  |  |  |
| ARVC-48 | No family history of ARVC | gene-elusive |  |  |  |
| ARVC-49 | No family history of ARVC | gene-elusive |  |  |  |
| ARVC-50 | No family history of ARVC | gene-elusive |  |  |  |
| ARVC-51 | No family history of ARVC | gene-elusive |  |  |  |
| ARVC-52 | First-degree relative (brother) SCD at 48. | gene-elusive |  |  |  |
| ARVC-53 | No family history of ARVC | gene-elusive |  |  |  |
| ARVC-54 | First-degree relative (father) SCD at 62. | gene-elusive |  |  |  |
| ARVC-55 | First-degree relative (father) SCD at 50. | gene-elusive |  |  |  |
| DCM-1 | No family history of DCM | *TTN* | c.72169+1G>C | p.(?) | Likely pathogenic |
| DCM-2 | No family history of DCM | *TTN* | c.37888del | p.(Ser12630fs) | Likely pathogenic |
| DCM-3 | No family history of DCM | *TTN* | c.55489_55492dup | p.(Asn18498*) | Likely pathogenic |
| DCM-4 | No family history of DCM | *TTN* | c.75929_75930del | p.(Thr25310fs) | Likely pathogenic |
| DCM-5 | No family history of DCM | *LMNA* | c.1702A>C | p.(Ser568Arg) | Likely pathogenic |
| DCM-6 | First-degree relative (sister) and second-degree relatives (grandmother and aunt (both on fathers' side)) with confirmed DCM | gene-elusive |  |  |  |
| DCM-7 | First-degree relative (sister) with confirmed DCM | Genotype unknown | unknown | unknown |  |
| DCM-8 | First-degree relative (brother) with confirmed DCM. | *LMNA* | c.777T>A | p.(Tyr259*) | Pathogenic |
| DCM-9 | First-degree relative (daughter and son) with confirmed DCM | gene-elusive |  |  |  |
| DCM-10 | No family history of DCM | *FLNC* | c.6485-2A>T | p.(?) | Likely pathogenic |
| DCM-11 | First-degree relative (father, no abnormalities found with genetic testing) with confirmed DCM . | *LMNA* | c.1146C>T | p.(Gly382*) | Likely pathogenic |
| DCM-12 | First-degree relative (son) with confirmed DCM | gene-elusive |  |  |  |
| DCM-13 | First-degree relative (mother) with confirmed DCM. | gene-elusive |  |  |  |
| DCM-14 | Third-degree relative (cousin) with confirmed DCM | *FLNC* | c.1948C>T | p.(Arg650*) | Likely pathogenic |
| DCM-15 | First-degree relative (mother) and second-degree relative (grandmother (mothers' side) with confirmed DCM | *ACTC1* | c.725A>G | p.(Tyr242Cys) | Likely pathogenic |
| DCM-16 | First-degree relative (mother) with confirmed DCM. | *TNNI3* | c.292C>T | p.(Arg98*) | Likely pathogenic |
| DCM-17 | No family history of DCM | gene-elusive |  |  |  |
| DCM-18 | First-degree relative (father) with confirmed DCM | *BAG3* | c.1087G>T | p.(Glu363*) | Likely pathogenic |
| DCM-19 | Second-degree relative (cousin) with confirmed DCM. | gene-elusive |  |  |  |
| DCM-20 | First-degree relative (sister) with confirmed DCM | *PLN* | c.40_42delAGA | p.(Arg14del) | Pathogenic |
| DCM-21 | First-degree relative (brother) with confirmed DCM | gene-elusive |  |  |  |
| DCM-22 | First-degree relative (mother) with confirmed DCM | gene-elusive |  |  |  |
| DCM-23 | First-degree relative (brother) with confirmed DCM; First-degree relative (sister) with SCD at age 29 | *TTN* | c.59820C>A | p.(Tyr19940*) | Pathogenic |
| DCM-24 | No family history of DCM | *DES* | c.854C>T | p.(Ala285Val) | Likely pathogenic |
| DCM-25 | First-degree relative (mother) with confirmed DCM | *TNNI3* | c.292C>T | p.(Arg98*) | Likely pathogenic |

Genetic characteristics of subjects with ARVC or DCM. Abbreviations: *ACTC1*, Actin alpha cardiac muscle-1; ARVC, arrhythmogenic right ventricular cardiomyopathy; *BAG3*, BCL2 associated athanogene 3; DCM, dilated cardiomyopathy; *DES*, Desmin; *DSG2*, Desmoglein-2; *FLNC*, Filamin-C; *TNNI3*, Troponin-I 3; *LMNA*, Lamin A/C; *MYH7*, Myosin Heavy Chain-7; *PKP2*, Plakophilin-2; *PLN*, Phospholamban; SCD, sudden cardiac death; TTN, Titin.

## **Table S2.** Intra- and interobserver variability

| **Measurement** | **Intra-observer variability** | **Interobserver variability** |
| --- | --- | --- |
| RV reproducibility | 0.83 | 0.71 |
| LV reproducibility | 0.82 | 0.79 |

Reproducibility measured by intra- and interobserver variability. Both RV and LV reproducibility assess simultaneously the presence of any LGE and, if LGE is present, the ability of the observer for discriminating between Padua fulfilling LGE and not Padua fulfilling LGE by weighted Cohen’s Kappa. All numbers represent Cohen’s Kappa. Abbreviations as in text.

## **Table S3.** Study population stratified by LGE per Padua-criteria

|  | Fulfilling both LV- and RV-LGE per Padua criteria (N=43) | Only fulfilling LV-LGE per Padua criteria (N=39) | Only fulfilling RV-LGE per Padua criteria (N=20) | Neither fulfilling LV nor RV-LGE per Padua criteria (N=30) | p-value |  |
| --- | --- | --- | --- | --- | --- | --- |
| **Diagnosis** |  |  |  |  | <0.001 |  |
| ARVC | 10 (23.3) | 13 (33.3) | 10 (50.0) | 22 (73.3) |  |  |
| DCM | 3 (7.0) | 15 (38.5) | 0 (0.0) | 7 (23.3) |  |  |
| Myocarditis | 9 (20.9) | 4 (10.3) | 0 (0.0) | 0 (0.0) |  |  |
| Sarcoidosis | 15 (34.9) | 5 (12.8) | 0 (0.0) | 0 (0.0) |  |  |
| Amyloidosis | 6 (14.0) | 2 (5.1) | 10 (50.0) | 1 (3.3) |  |  |
| **Demographics** | | | | | | |
| Age (yrs) | 48±18 | 48±15 | 52±18 | 41±20 | 0.186 |  |
| Male sex (%) | 29 (67.4) | 24 (61.5) | 13 (65.0) | 13 (43.3) | 0.195 |  |
| **Global CMR parameters** | | | | | | |
| *LV CMR parameters* | | | | | | |
| EF (%) | 52 [41-57] | 46 [24-54] | 54 [44-56] | 52 [46-55] | 0.054 |  |
| EDV/BSA | 100 [80-113] | 106 [96-161] | 98 [88-106] | 99 [88-114] | 0.035 |  |
| ESV/BSA | 46 [34-61] | 59 [45-111] | 43 [38-63] | 49 [41-57] | 0.020 |  |
| SV | 88±26 | 90±34 | 95±21 | 96±22 | 0.594 |  |
| *RV CMR parameters* |  |  |  |  |  |  |
| EF (%) | 47 [38-54] | 44 [35-52] | 48 [43-51] | 50 [47-57] | 0.023 |  |
| EDV/BSA | 104±27 | 102±27 | 100±20 | 96±25 | 0.693 |  |
| ESV/BSA | 51 [41-74] | 53 [44-79] | 53 [43-63] | 50 [38-61] | 0.576 |  |
| SV | 85±28 | 82±31 | 90±18 | 90±24 | 0.575 |  |
| **LGE paramaters** | | | | | | |
| *LV LGE* | | | | | | |
| Median number of LV-LGE segments | 9 [5-12] | 5 [2-7] | 10 [0-17] | 0 [0-0] | <0.001 |  |
| LV-LGE present | 43 (100.0) | 39 (100.0) | 11 (55.0) | 5 (16.7) | <0.001 |  |
| Anterior | 22 (51.2) | 11 (28.2) | 10 (50.0) | 1 (3.3) | <0.001 |  |
| Basal  anterior | 17 (39.5) | 8 (20.5) | 10 (50.0) | 1 (3.3) | <0.001 |  |
| Mid anterior | 19 (44.2) | 8 (20.5) | 10 (50.0) | 1 (3.3) | <0.001 |  |
| Anteroseptal | 28 (65.1) | 19 (48.7) | 10 (50.0) | 1 (3.3) | <0.001 |  |
| Basal anteroseptal | 25 (58.1) | 17 (43.6) | 10 (50.0) | 1 (3.3) | <0.001 |  |
| Mid anteroseptal | 22 (51.2) | 11 (28.2) | 10 (50.0) | 1 (3.3) | <0.001 |  |
| Anterolateral | 27 (62.8) | 18 (46.2) | 10 (50.0) | 1 (3.3) | <0.001 |  |
| Basal anterolateral | 19 (44.2) | 11 (28.2) | 10 (50.0) | 1 (3.3) | <0.001 |  |
| Mid anterolateral | 24 (55.8) | 12 (30.8) | 10 (50.0) | 1 (3.3) | <0.001 |  |
| Inferior | 30 (69.8) | 15 (38.5) | 10 (50.0) | 1 (3.3) | <0.001 |  |
| Basal inferior | 25 (58.1) | 11 (28.2) | 10 (50.0) | 1 (3.3) | <0.001 |  |
| Mid inferior | 23 (53.5) | 13 (33.3) | 10 (50.0) | 1 (3.3) | <0.001 |  |
| Inferoseptal | 31 (72.1) | 25 (64.1) | 10 (50.0) | 1 (3.3) | <0.001 |  |
| Basal inferoseptal | 25 (58.1) | 21 (53.8) | 10 (50.0) | 1 (3.3) | <0.001 |  |
| Mid inferoseptal | 29 (67.4) | 19 (48.7) | 10 (50.0) | 1 (3.3) | <0.001 |  |
| LGE inferolateral | 34 (79.1) | 20 (51.3) | 10 (50.0) | 1 (3.3) | <0.001 |  |
| Basal inferolateral | 26 (60.5) | 17 (43.6) | 10 (50.0) | 1 (3.3) | <0.001 |  |
| Mid inferolateral | 28 (65.1) | 15 (38.5) | 10 (50.0) | 1 (3.3) | <0.001 |  |
| Apex | 31 (72.1) | 16 (41.0) | 11 (55.0) | 1 (3.3) | <0.001 |  |
| Apical anterior | 15 (34.9) | 9 (23.1) | 10 (50.0) | 1 (3.3) | 0.001 |  |
| Apical septal | 21 (48.8) | 8 (20.5) | 11 (55.0) | 1 (3.3) | <0.001 |  |
| Apical inferior | 18 (41.9) | 7 (17.9) | 11 (55.0) | 1 (3.3) | <0.001 |  |
| Apical lateral | 22 (51.2) | 13 (33.3) | 10 (50.0) | 1 (3.3) | <0.001 |  |
| True apex | 14 (32.6) | 4 (10.3) | 10 (50.0) | 1 (3.3) | <0.001 |  |
| *RV LGE* | | | | | | |
| Median number of RV-LGE segments | 4 [2-6] | 0 [0-0] | 6 [3-7] | 0 [0-0] | <0.001 |  |
| RV-LGE present | 43 (100.0) | 3 (7.7) | 20 (100.0) | 0 (0.0) | <0.001 |  |
| Basal LGE | 32 (74.4) | 0 (0.0) | 16 (80.0) | 0 (0.0) | <0.001 |  |
| Basal inferior | 25 (58.1) | 0 (0.0) | 13 (65.0) | 0 (0.0) | <0.001 |  |
| Basal lateral | 30 (69.8) | 0 (0.0) | 13 (65.0) | 0 (0.0) | <0.001 |  |
| Mid LGE | 33 (76.7) | 3 (7.7) | 17 (85.0) | 0 (0.0) | <0.001 |  |
| Mid inferior | 24 (55.8) | 1 (2.6) | 14 (70.0) | 0 (0.0) | <0.001 |  |
| Mid lateral | 28 (65.1) | 3 (7.7) | 17 (85.0) | 0 (0.0) | <0.001 |  |
| Apical LGE | 28 (65.1) | 0 (0.0) | 15 (75.0) | 0 (0.0) | <0.001 |  |
| Apical inferior | 24 (55.8) | 0 (0.0) | 14 (70.0) | 0 (0.0) | <0.001 |  |
| Apical lateral | 18 (41.9) | 0 (0.0) | 12 (60.0) | 0 (0.0) | <0.001 |  |
| RV outflow tract | 21 (48.8) | 0 (0.0) | 12 (60.0) | 0 (0.0) | <0.001 |  |
| *Miscellaneous segments* | | | | | | |
| Atrial LGE | 12 (27.9) | 4 (10.3) | 10 (50.0) | 0 (0.0) | <0.001 |  |
| Valvular LGE | 4 (9.3) | 3 (7.7) | 9 (45.0) | 1 (3.3) | <0.001 |  |
| Papillary muscle LGE | 11 (25.6) | 2 (5.1) | 10 (50.0) | 0 (0.0) | <0.001 |  |
| Moderator band LGE | 11 (25.6) | 0 (0.0) | 10 (50.0) | 0 (0.0) | <0.001 |  |
| Whale’s Tail sign | 11 (25.6) | 2 (5.1) | 0 (0.0) | 0 (0.0) | <0.001 |  |
| LGE in any one of the above | 22 (51.2) | 8 (20.5) | 10 (50.0) | 1 (3.3) | <0.001 |  |

A p-value of < 0.001 was considered statistically significant in this table after Bonferroni correction Abbreviations: ARVC, Arrhythmogenic right ventricular cardiomyopathy; BSA, Body surface area; CMR, Cardiac magnetic resonance imaging; EDV, End-diastolic volume; EF, Ejection fraction; ESV, End-systolic volume; LGE, Late gadolinium enhancement; LV, Left ventricular; RV, Right ventricular; SV, stroke volume; yrs, years.

# **Supplementary Figures**

## **Figure S1.** Ventricular segmentation


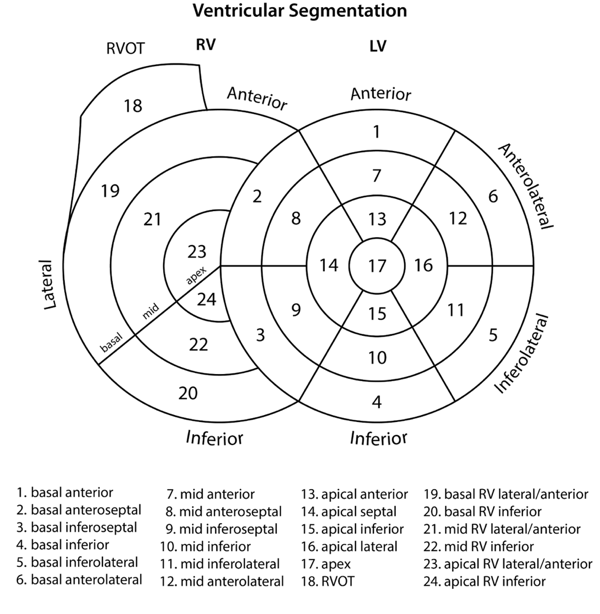


Segmentation of the LV and RV. All segments are denoted by numbers which are correspond with the legend in the figure. Abbreviations as in text.

## **Figure S2.** Whale’s Tail sign in a patient with sarcoidosis


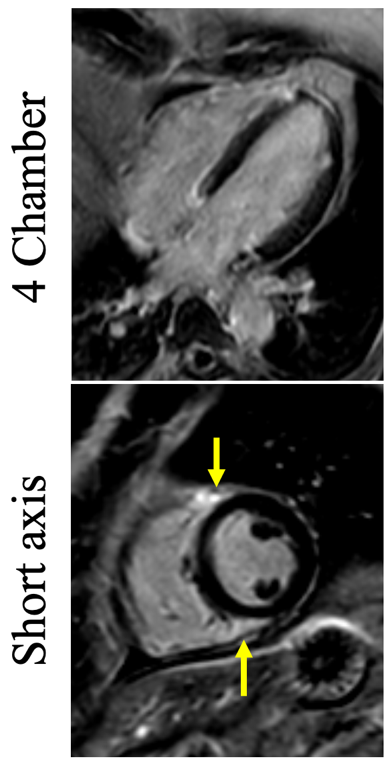


Patient with FDG-PET and biopsy confirmed cardiac sarcoidosis showing subepicardial LGE in the LV anterior and inferior wall extending into the septal wall and RV wall (whale’s tail sign; arrows)

## **Figure S3.** Presence of RV-LGE per Padua Criteria


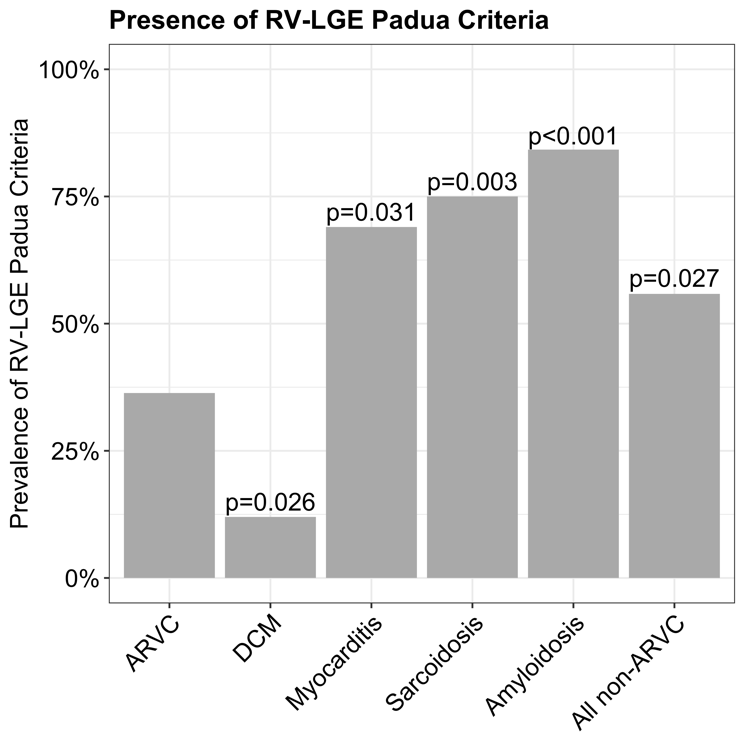


Presence of LV-LGE per Padua criteria (y-axis) stratified by phenotype (x-axis). Statistical difference of LV-LGE presence is denoted on top of every bar.

## **Figure S4.** Distribution pattern of LGE for “genetically proven ARVC”-group


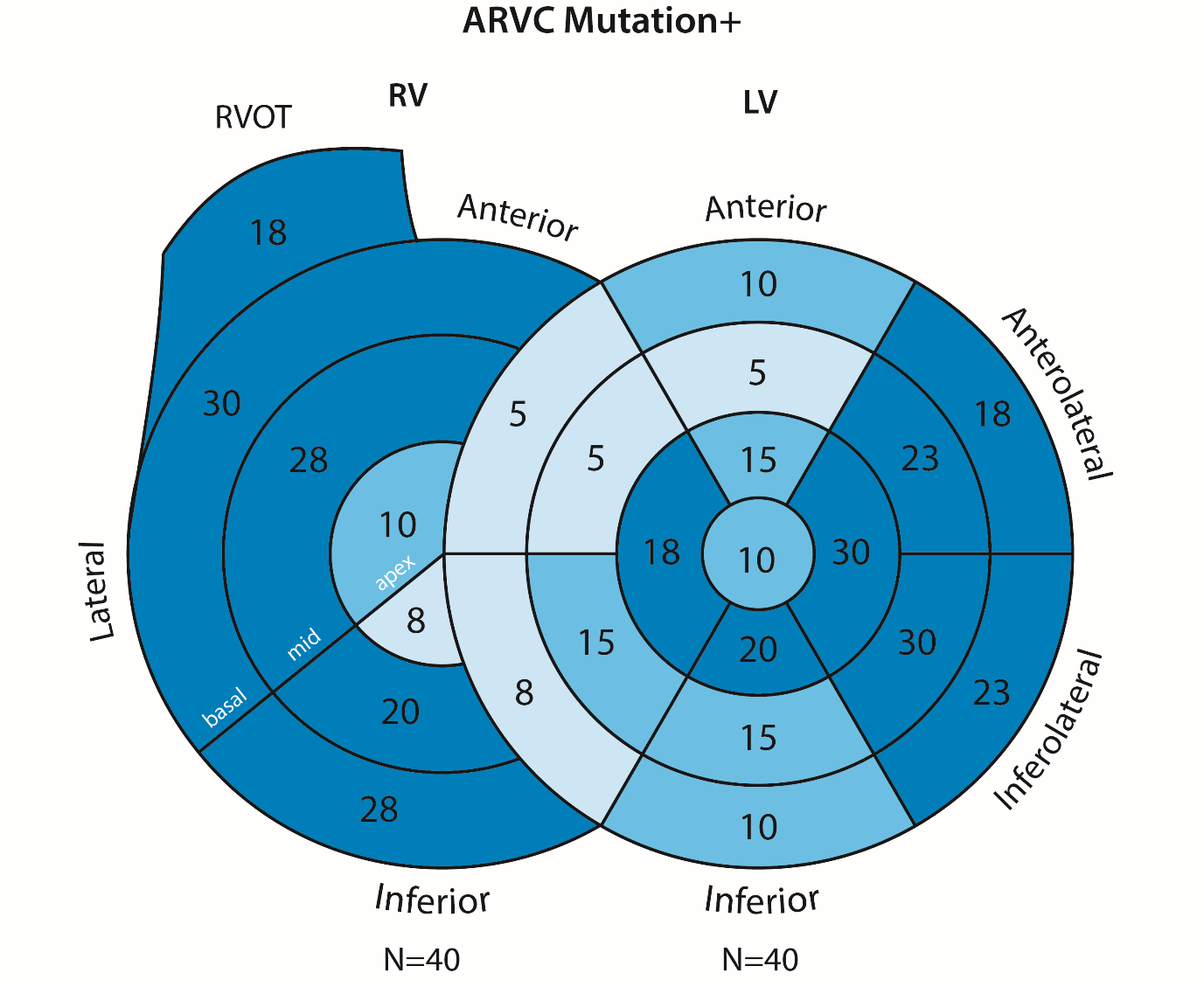
Overview of LGE distribution. Numbers per segment represent percentages. Color representation same as in Figure 2. Abbreviations as in text.

## **Figure S5.** Prevalence and diagnostic value of RV-LGE and LV-LGE per Padua criteria for “genetically proven ARVC”-group and its differentials.


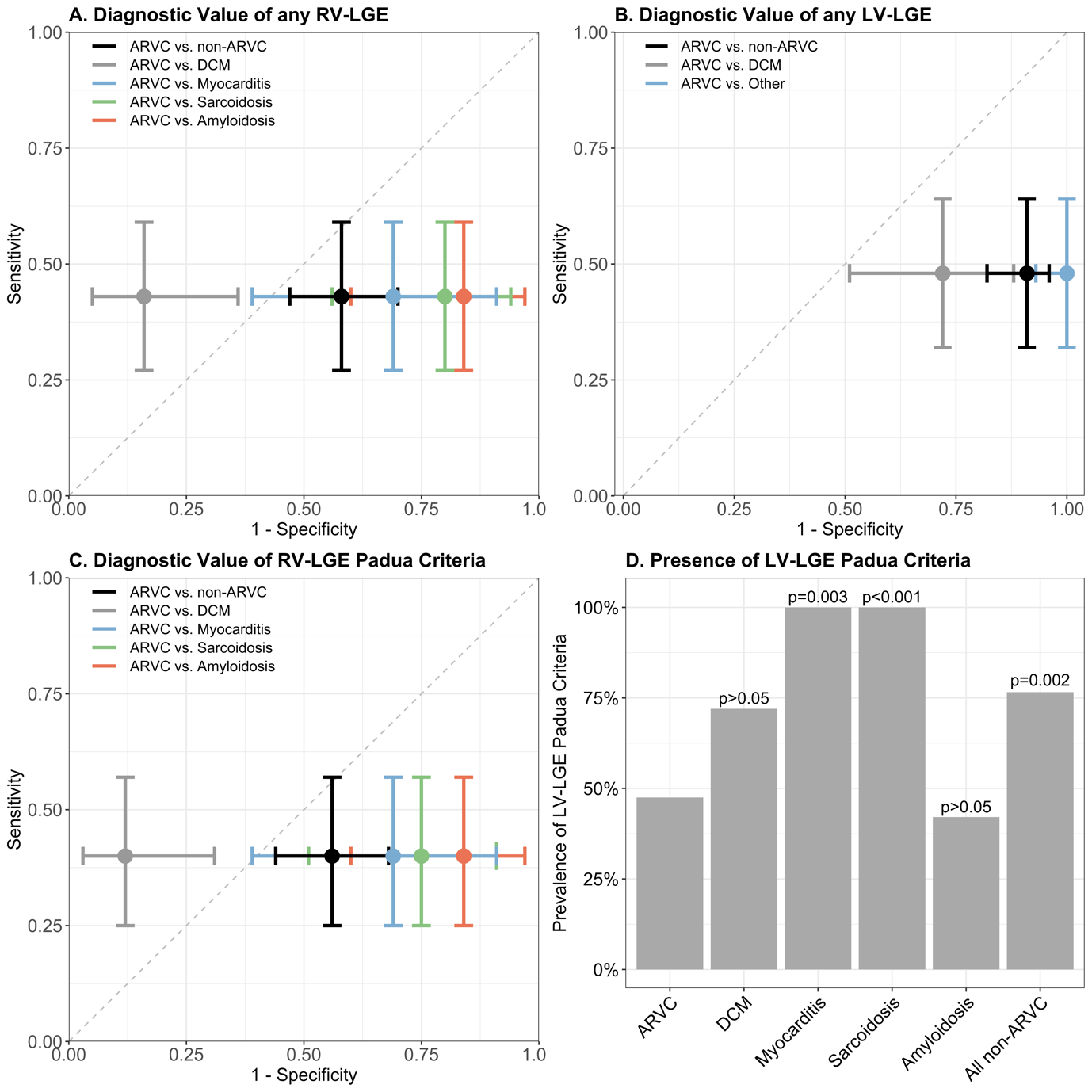


Sensitivity (y-axis) and 1-specificity (x-axis) of (**A**) any RV-LGE and (**B**) any LV-LGE to differentiate between “genetically proven ARVC”-group and non-ARVC (black), DCM (grey), myocarditis (blue), sarcoidosis (green), and amyloidosis (orange). (**C**) Sensitivity (y-axis) and 1-specificity (x-axis) of RV-LGE per Padua criteria to differentiate between ARVC and non-ARVC (black), DCM (grey), and a composite of myocarditis, sarcoidosis, and amyloidosis (light blue). Myocarditis, sarcoidosis, and amyloidosis had all a specificity of 0% and a sensitivity of 34% and were therefore visualized as a composite measure. The grey dashed line in figure A-C indicates the line of no discrimination. Since LGE was described as a binary variable, dots and error bars (denoting 95% confidence intervals) are shown, instead of a line graph. A dot in the left-upper side of the line of no discrimination favours a positive association of the variable with ARVC diagnosis, whereas a dot in the right-lower side of the line of no discrimination favours a negative association of the variable with ARVC diagnosis. (**D**) Presence of LV-LGE per Padua criteria (y-axis) stratified by diagnosis (x-axis). Statistical difference of LV-LGE presence is denoted on top of every bar
